# Introducing the D‐DAND scale: Development of a comprehensive caregiver‐administered tool for Dravet syndrome comorbidities

**DOI:** 10.1002/epi.70399

**Published:** 2026-08-18

**Authors:** Bernardo Dalla Bernardina, Francesca Offredi, Alessio Toraldo, Lisa Ouss, Delphine Breuillard, Mathilde Cozzo, Tommaso Lo Barco, Isabella Brambilla, Francesca Darra, Rima Nabbout

**Affiliations:** ^1^ Research Center for Pediatric Epilepsies Azienda Ospedaliera Universitaria Integrata of Verona Verona Italy; ^2^ Child Neuropsychiatry Unit Azienda Ospedaliera Universitaria Integrata of Verona, full member of the European Reference Network EpiCARE Verona Italy; ^3^ Department of Brain and Behavioral Sciences University of Pavia Pavia Italy; ^4^ Milan Center for Neuroscience Milan Italy; ^5^ Reference Center for Rare Epilepsies, Department of Pediatric Neurology, Necker Enfants Malades Hospital, APHP, full member of the European Reference Network EpiCARE Université Paris Cité Paris France; ^6^ Fondazione Dravet ETS member of the European Reference Network EpiCARE, Patient Advocates Group Verona Italy; ^7^ Child Neuropsychiatry Section, Department of Engineering for Innovation Medicine University of Verona Verona Italy; ^8^ Institut Imagine, Inserm U1163 Paris France

**Keywords:** Dravet Disease‐Associated Neuropsychiatric Disorders (D‐DAND), Dravet syndrome, neuropsychiatric assessment, neuropsychiatric outcomes

## Abstract

**Objective:**

The Dravet Disease‐Associated Neuropsychiatric Disorders (D‐DAND) scale is a new caregiver‐administered interview designed to assess the wide range of developmental and behavioral comorbidities in Dravet syndrome (DS) beyond seizures. D‐DAND's preliminary psychometric properties in a large patient sample are reported.

**Methods:**

D‐DAND was developed by a panel of experts and later standardized with caregivers of 123 individuals with DS (age = 3–41 years); a subsample of 43 caregivers were interviewed twice (~2 weeks apart) to evaluate test–retest reliability; another subsample of 80 were also administered with other scales that are widely used despite not being tailored to DS (Vineland Adaptive Behavior Scales, Child Behavior Checklist, Childhood Autism Rating Scale, Behavior Rating Inventory of Executive Function, Sleep Disturbance Scale for Children).

**Results:**

The D‐DAND captured a broad spectrum of abilities and dysfunctions across multiple functional domains. Test–retest reliability was excellent (overall score: *r* ≈ .98, median across domains: *r* ≈ .89), thus allowing for high sensitivity to time‐related changes. As expected, D‐DAND scores showed high correlations with some standardized measures (e.g., Vineland scales) and low correlation with others, suggesting that D‐DAND measures unique, Dravet‐specific features that other, widely used tools fail to detect.

**Significance:**

D‐DAND is a novel, reliable tool for comprehensive evaluation of DS beyond seizures. It enables systematic tracking of developmental and behavioral issues in DS, facilitating early identification of needs and more personalized, whole‐person care.

## Graphical abstract

Graphical overview of the Dravet Disease–Associated Neuropsychiatric Disorders (D‐DAND) scale. The D‐DAND scale provides a caregiver‐based, comprehensive assessment of developmental and behavioral comorbidities in Dravet syndrome across six domains: motor abilities, language and social interaction, autonomies, academic skills, emotional/behavioral regulation, and sleep. Domain scores contribute to an overall score, enabling systematic characterization and longitudinal monitoring of patient functioning beyond seizures.

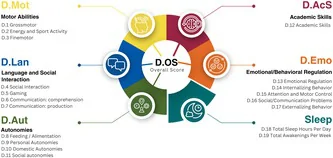

**Key points**

- D‐DAND is the first Dravet syndrome‐specific scale assessing developmental, behavioral, and daily life issues beyond seizures.
- In a multicenter study (*N* = 123), D‐DAND showed excellent test–retest reliability (*r* = .98 for the overall score) over a 2‐week interval.
- D‐DAND scores correlated with some standardized measures (adaptive behavior, autism features, etc.).
- D‐DAND captured unique Dravet‐specific problems (e.g., gait and sleep difficulties) that generic scales failed to detect.

## 1 INTRODUCTION

Dravet Syndrome (DS) is a prototypical monogenic developmental and epileptic encephalopathy. DS relates to pathogenic variants in *SCN1A* in the majority of patients. Seizures occur in the first year of life in otherwise healthy infants; they are often febrile, generalized tonic–clonic seizures or unilateral clonic seizures. Seizures are also often prolonged and evolve to status epilepticus. Many patients later develop other seizure types, like myoclonic seizures, atypical absences, and focal seizures.^1^, ^2^ Developmental delay becomes apparent within the second year of life, evolving into definite cognitive impairment and behavioral and psychiatric disorders such as hyperactivity, attention deficit, opposition behavior and, less often, autistic traits and other psychiatric disorders.^2^, ^3^, ^4^, ^5^, ^6^ These disorders cannot be trivially interpreted as direct consequences of epilepsy.^7^, ^8^ Genetic mutations have a definite role in these comorbidities. Yet, the relative contribution of epileptic activity and antiseizure medications (ASMs) to cognitive and behavioral trajectories is still undefined. In particular, the influence of seizure severity and recurrence, together with ASM exposure, on long‐term nonseizure outcomes remains a major knowledge gap.^9^

Although seizures tend to decrease in frequency and severity between adolescence and adulthood, cognitive, behavioral, and motor impairments have worsened over time in several subjects.^4^, ^10^ Moreover, other issues like sleep and feeding disorders are often overlooked.^11^, ^12^, ^13^, ^14^ These nonseizure features substantially shape the long‐term outcome for patients and families.^9^, ^15^, ^16^, ^17^, ^18^ Although associating drug‐resistant epilepsy and other morbidities, the DS course is characterized by interindividual and intraindividual variability. Consequently, every subject needs a systematic and periodic comprehensive assessment.^19^ Despite these needs, a holistic assessment tool tailored to the specific features of DS patients is still missing. Other scales—not tailored to this population—are “borrowed” from other fields of intervention (e.g., Vineland Adaptive Behavior Scales (VABS), Child Behavior Checklist (CBCL), Childhood Autism Rating Scale (CARS), Behavior Rating Inventory of Executive Function (BRIEF)) but prove to be insufficient to cover the whole spectrum of the DS clinical–behavioral issues. Another drawback of such scales is that they are often tailored to the normal population; DS patients' severe intellectual impairment leads to pronounced “floor” or “ceiling” effects, thus missing relevant functioning differences or time‐related evolution.^20^, ^21^ Furthermore, these scales are time‐consuming and have limited repeatability, as they cannot be systematically combined with the electroclinical assessments required for adequate follow‐up monitoring. Tools like (e.g.) DANCE focus on screening but do not evaluate deficit severity; neither can they detect slight changes in the severe‐to‐profound deficit range.^22^

To fill this gap, we developed a tool that is specific for the Dravet population. The Dravet Disease‐Associated Neuropsychiatric Disorders (D‐DAND) scale is a caregiver‐based structured interview assessing the everyday functional domains that are most relevant to families and clinicians—adaptive skills, communication, motor abilities, behavioral/emotional problems, and sleep/feeding—thereby complementing seizure characterization and enabling sensitive longitudinal monitoring, avoiding floor/plateau effects. We developed the scale so as to provide deficit severity estimates that are well known in clinical settings, that is, *z*‐scores, and also studied their test–retest reliability to provide indicators of significant changes across time. Our patients were also tested with the scales that are commonly used for quantifying their behavioral features (“external scales”) but that are not tailored to the Dravet population, predicting that the correlation with D‐DAND would be suboptimal.

## 2 MATERIALS AND METHODS

### 2.1 Development of the D‐DAND

The target of the early phases was that of identifying the main features of the clinical/cognitive/behavioral profiles of Dravet patients. An international expert panel (France/Italy; three child neurologists, one psychiatrist, two neuropsychologists, and one family representative) iteratively refined the content of the scale across five discussion rounds (May 2018–May 2020), focusing on clinical relevance, clarity, and expected sensitivity to change (Figure S1). The experts agreed on a large set of features to be measured in Dravet subjects and that these items should cover areas of practical significance—areas of intervention—rather than areas of neurocognitive impairment. These areas were called “domains” and were further divided into “subdomains” (see Figure 1). The specific contents of domains, subdomains, and items were decided on the grounds of a thorough review of the relevant literature and on the clinical features that are characteristic of DS, as determined by expert clinical experience and by input from parents. This ensured coverage of functionally meaningful everyday outcomes.

**FIGURE 1:**
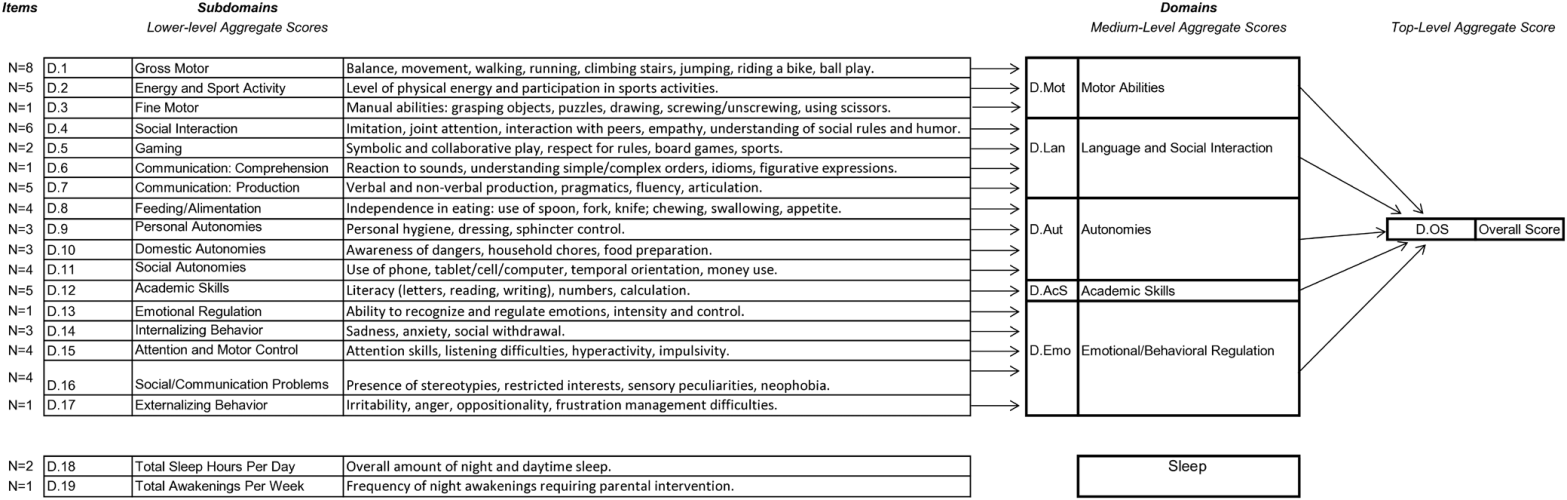
Hierarchical structure of the D‐DAND (Dravet Disease‐Associated Neuropsychiatric Disorders) scale. Items are grouped into 19 “subdomains,” which in turn are grouped into five “domains”: D.Mot (Motor Abilities), D.Lan (Language and Social Interaction), D.Aut (Autonomies), D.AcS (Academic Skills), and D.Emo (Emotional/Behavioral Regulation). Scores are aggregated through this hierarchy, up to an overall score, D.OS (Overall Score).

Early versions of the D‐DAND were checklists; from version 5 (April 2020) on, the tool was reorganized into two parallel components: a Neurodevelopment section (competence‐based ratings; seven domains and 41 subdomains, with 4–7 competence levels) and a Follow‐up section (34 subdomains, including Emotional/Behavioral Problems) intended to capture short‐term qualitative fluctuations. Pilot testing (June–October 2020) in caregivers of 12 Italian patients (mean age = 9.6 years) and four French patients (mean age = 9 years) generated structured feedback from caregivers and clinicians as to length, relevance, clarity, and need for examples. Following three additional core‐group refinement rounds (October 2020–April 2021), the scale was consolidated into a single comprehensive instrument integrating developmental profiling and longitudinal qualitative change, harmonizing item directionality, and finalized with translation/back‐translation procedures for modified items.

### 2.2 Final version of D‐DAND

The final version included 63 questions (“items”), in most of which (60/63, belonging to subscales D.1–D.17), caregivers provide qualitative descriptions of the patient's current functioning, and the examiner checks one of the levels of a closed, 0–100 ordinal scale. Items vary for the number of levels (3–7, including 0 and 100). Higher scores consistently reflect better competence in acquired skills or lower severity/frequency/impact of difficulties; thus, 0 represents absence of competence (maximal impairment), 100 full competence (no impairment). Zero to 100 scores from different items are then averaged, producing aggregate scores. Three hierarchical levels of aggregation are applied, with the intermediate levels corresponding to the “subdomain” and “domain” levels mentioned above. Thus, the initial 60 items are aggregated (1) into 19 “subdomain” scores, then (2) into five, broader “domain” scores, and finally (3) into a single, global score (see Figure 1). The five domains—Motor Abilities, Language and Social Interaction, Autonomy, Academic Skills, and Behavioral and Emotional Problems—summarize the main areas of functioning relevant to longitudinal monitoring in DS. Items related to sleep are treated separately, because their scales are not closed.

Some examples of items follow:

- Motor competence example (D.1, Running)
  ⚬ 0: Inability to run
  ⚬ 25: Runs with a trotting gait
  ⚬ 50: Runs but with some falls
  ⚬ 75: Runs without falling, but has difficulty changing direction
  ⚬ 100: Runs in a coordinated way and changes direction without falling
- Problem severity example (D.15, Listening and Attentional Difficulties)
  ⚬ 0: Does not listen to instructions, does not seem to listen when spoken to (not related to difficulties understanding instructions), even when not engaged in an activity
  ⚬ 25: Does not listen to instructions, does not seem to listen when spoken to (not related to comprehension difficulties) when engaged in or doing an activity
  ⚬ 50: Seems elsewhere when spoken to, often asks for instructions or to repeat what was just said
  ⚬ 75: Listens to instructions except those given a few minutes earlier
  ⚬ 100: Listens to and follows instructions appropriately, including those given a few minutes earlier

### 2.3 Participants

We recruited 123 patients with confirmed DS diagnosis and age ≥ 3 years, at two specialized centers, one in Italy and one in France. Informed consent was obtained from caregivers. The sample included children (preschool and school age), adolescents, and a subset of adults. Despite the wide age range, most patients exhibited significant developmental impairments, consistent with moderate to severe intellectual disability (e.g., low VABS‐II adaptive scores; see later) and a high prevalence of autism and attention‐deficit/hyperactivity disorder symptoms (per clinical notes). Eighty patients (subsample 1: 39 males, mean age = 13.6 years, range = 3–41) joined a first study, in which caregivers were administered a single, comprehensive assessment including both D‐DAND and a set of other, standardized scales (henceforth, “external” scales) that are often used in clinical practice but which are not tailored to the DS population. Forty‐three patients (subsample 2: 28 males, mean age = 15.6 years, range = 3–39) received only D‐DAND, but twice, an average of 2 weeks apart. This provided an estimate of D‐DAND's test–retest reliability, which in turn was necessary to set the limits of a statistically significant D‐DAND score change. The final D‐DAND standardization was performed on the whole *N* = 123 set, whose demographic details are reported in Table 1.

**TABLE 1:** Demographics of the Dravet syndrome patients whose caregivers underwent the D‐DAND interview.

Subsample 1	Nat	Sex	*N*	Age (months)			
				Mean	SD	Min	Max
	FR	F	19	118.1	58.0	43	228
	FR	M	18	131.6	58.1	37	233
	IT	F	22	223.9	107.2	77	495
	IT	M	21	169.7	91.0	46	465
	Overall		80	163.8	91.6	37	495

Subsample 2	Nat	Sex	*N*	Age (months)			
				Mean	SD	Min	Max
	FR	F	9	149.0	69.2	55	274
	FR	M	18	155.2	106.7	36	459
	IT	F	6	195.7	144.2	40	398
	IT	M	10	276.0	138.1	50	470
	Overall		43	187.7	121.4	36	470

*Note*: Subsample 1 received D‐DAND once, but was also administered, additional, “external” scales; subsample 2 received D‐DAND twice, an average of 2 weeks apart.

Abbreviations: D‐DAND, Dravet Disease‐Associated Neuropsychiatric Disorders; F, female; FR, French; IT, Italian; M, male; Max, maximum; Min, minimum; Nat, Nationality; SD, Standard Deviation.

Given the rarity of DS, we capitalized on the bi‐sites collaboration with the families' advocacy groups in both countries and could cover a wide range of ages; however, older patients mostly belonged to the Italian subgroups (especially in subsample 1), hence the need for careful covariation (by General Linear Models [GLMs]; see Statistical Analyses).

### 2.4 Procedure

The caregiver sat in a quiet room and was asked the questions orally in the national language. The D‐DAND interview took an average of ~35 min (minimum–maximum = 15–60). In subsample 1, D‐DAND was followed by the administration of the external scales; this occurred within the same session as, or within a week of, D‐DAND. In subsample 2, the two D‐DAND administrations occurred an average of 2 weeks apart (precisely, 13.7 days; minimum–maximum = 6–25).

### 2.5 External scales

We expected the external scales to correlate with the D‐DAND scale in a variegated way, ranging from high to low correlations, and in general in a suboptimal way, because external scales were developed for completely different purposes and were not tailored to the Dravet population. We selected external scales' versions that were validated in both French and Italian, and which approximated the behaviors described by specific D‐DAND subdomains as closely as possible. The external scales coupled with D‐DAND subdomains are reported in Table 5.

- Motor/adaptive skills: VABS‐II,^23^, ^24^ interview form; caregiver's survey of adaptive functioning (communication, daily living, socialization, motor behavior): VABS‐II items were coupled with D‐DAND's motor and adaptive skill domains (subdomains D.1, D.11; Table 1).
- Executive functions: Behavior Rating Inventory of Executive Function (BRIEF),^25^, ^26^, ^27^, ^28^, ^29^ parent forms (versions: Preschool, Child, Adult) evaluating executive functioning in everyday life: These different BRIEF versions were coupled with D‐DAND attentional and executive‐related subdomains, D.13, D.15, D.16.
- Emotional–behavioral difficulties: Child Behavior Checklist (CBCL),^30^, ^31^, ^32^ a broad caregiver‐rated measure of emotional and behavioral problems (with T‐score outputs for internalizing, externalizing, etc.): CBCL items were coupled with D‐DAND's behavioral/emotional subdomains, D.14, D.15, D.17.
- Autism‐related behavior: Childhood Autism Rating Scale (CARS),^33^, ^34^ a clinician/caregiver tool for identifying autism spectrum disorder features. CARS scores were coupled with two D‐DAND scores related to autistic symptoms, D.4, D.16.

### 2.6 Statistical analyses

The D‐DAND project aimed at quantifying deficit severity in terms of *z*‐scores, because these are well known in clinical settings, allow for assessing worsening/improvement across the whole scale (differently from percentiles), and allow for straightforward statistical detection of changes with time. *Z*‐scores provide the abnormality of a score with respect to subjects with the same general demographics: thus, GLMs estimating the effects of Age, Sex, and Nationality were necessary for obtaining *z*‐scores. Age and Sex are known to influence neurocognitive and behavioral variables, and Nationality was included to detect possible, subtle differences in the interpretation of the questions in the two different languages. The several steps leading to *z*‐scores are detailed below. All analyses were performed by means of JAMOVI (2019).^35^

#### 2.6.1 Creating aggregate 0–1 scales out of single‐item scores

Given that most items shared the same 0–100 scale, they could be averaged, obtaining *aggregate* scores. First, we rescaled each 0–100 variable to a 0–1 *proportion* variable (for mathematical reasons; see later). Scores belonging to the same subdomain (Figure 1) were then averaged, a procedure that led to 17 aggregate scores, D.1–D.17. These 17 were further merged to produce five scores, those of the “domain” level: D.Mot, D.Lan, D.Aut, D.AcS, and D.Emo (Figure 1). The five domain‐level aggregate scores, in turn, were merged into a single, overall score, D.OS. By “merged” we mean that when (e.g.) subdomains D.4, D.5, D.6, D.7 gave rise to D.Lan (Figure 1), the latter was the average of the 14 original items involved in D.4, D.5, D.6, D.7, and not the average of the four aggregate scores D.4, D.5, D.6, D.7 themselves. This choice preserved an item‐level‐equal‐weight approach, ensuring that each original item contributed equally to the merged score. Averaging aggregate subdomain scores would instead have assigned equal weight to subdomains with different numbers of items, thereby giving disproportionate influence to shorter, and thus less statistically stable, subdomains. As for the remaining four D‐DAND items, concerning sleep, two of them—hours slept during nighttime and daytime—were summed, producing the number of hours slept in a day. This is a 0–24 closed scale, which was thus also transformed into 0–1 (D.18). Another item concerned the number of weekly awakenings during nighttime sleep, an open scale (D.19). Descriptives for all D‐DAND 0–1 scores are reported in Table 2.

**TABLE 2:** D‐DAND standardization study on the full sample (*N* = 123).

		Descriptives					GLM predictors [parameters]					
		Scale range	Mean	SD	Min	Max	[SD_res_]	[Intercept]	*ln*(Age)	Sex	Nat.	Sex‐by‐Nat.
									[*β*_A_]	[*β*_S_]	[*β*_N_]	[*β*_SN_]
D.1	Grossmotor	0–1	.660	.187	.155	.969	1.001	.822	.240	−.337	.233	.505
D.2	Energy and Sport Activity	0–1	.703	.282	.000	1.000	2.192	1.655	−.**786**	−.379	**1.540**	1.005
D.3	Finemotor	0–1	.714	.211	.147	1.000	1.576	1.355	.**569**	.085	.195	.547
D.4	Social Interaction	0–1	.595	.219	.042	1.000	1.294	.527	−.035	.198	.397	.679
D.5	Gaming	0–1	.497	.292	.000	1.000	2.175	.153	−.021	.496	.758	**1.944**
D.6	Communication: comprehension	0–1	.665	.197	.250	1.000	1.671	1.147	.033	.198	−.**905**	.265
D.7	Communication: production	0–1	.688	.218	.100	1.000	1.436	1.089	.096	.171	−.129	**1.247**
D.8	Feeding / Alimentation	0–1	.778	.149	.333	1.000	1.406	1.667	−.062	−.012	−.429	.296
D.9	Personal Autonomies	0–1	.599	.282	.000	1.000	1.990	.774	.**753**	.278	−.541	1.387
D.10	Domestic Autonomies	0–1	.386	.238	.000	1.000	1.719	−.704	.370	.144	.430	**1.628**
D.11	Social autonomies	0–1	.330	.234	.000	1.000	1.431	−.957	.**749**	−.044	−.044	.794
D.12	Academic Skills	0–1	.412	.343	.000	1.000	2.494	−.706	**1.055**	−.021	.173	.677
D.13	Emotional Regulation	0–1	.563	.310	.000	1.000	2.562	.658	−.677	.613	.740	.286
D.14	Internalizing Behavior	0–1	.814	.183	.083	1.000	1.620	2.107	−.**761**	−.171	−.**761**	−.046
D.15	Attention and Motor Control	0–1	.550	.204	.125	1.000	1.020	.275	.**543**	.**624**	−.010	.184
D.16	Social/Communication Problems	0–1	.727	.216	.063	1.000	1.537	1.428	−.**459**	.488	−.**922**	.141
D.17	Externalizing Behavior	0–1	.413	.273	.000	1.000	2.144	−.490	−.608	.576	−.543	−1.455
D.18	Total Sleep Hours Per Day	0–24	10.359	1.484	6.500	14.000	.240	−.279	−.**125**	.044	.062	.056
D.19	Total Awakenings Per Week	0‐	1.325	2.627	.000	17.500						
D.Mot	Motor Abilities	0–1	.682	.176	.159	.982	1.017	.941	.283	−.202	.268	.514
D.Lan	Language and Social Interaction	0–1	.619	.193	.071	1.000	1.076	.610	−.025	.202	.136	.**821**
D.Aut	Autonomies	0–1	.528	.182	.131	.950	.826	.140	.**295**	.031	−.041	.501
D.AcS	Academic Skills	0–1	.412	.343	.000	1.000	2.494	−.706	**1.055**	−.021	.173	.677
D.Emo	Emotional/Behavioral Regulation	0–1	.656	.130	.301	.923	.600	.703	−.086	.**293**	−.**241**	.008
D.OS	Overall Score	0–1	.603	.153	.228	.893	.699	.471	.158	.046	−.017	.417

*Note*: Scales ranges and descriptives are reported in the left half of the table; the right half reports statistics from the GLM analysis performed on logit‐transformed D‐DAND scores. Regression parameters following Equation 1 (SD_res_, intercept, slopes *β*) are reported, as well as the predictors associated with slopes. Slopes are highlighted in bold when two‐tailed (*p* = .05) significant. *ln*(Age): positive slope = increase of score with log‐age. Sex: positive slope = higher score for female over male patients; Nat: positive = higher score for French over Italian patients. Sex by Nat: positive = French female patients show an extra advantage, over and above the effects of Sex and Nat. GLM parameters are not reported for D.19, because a different analysis was performed on it (see text). Abbreviations: D‐DAND, Dravet Disease‐Associated Neuropsychiatric Disorders; GLM, general linear model; Max, maximum; Min, minimum; Nat, Nationality;SD, Standard Deviation.

As for “external scales,” most of them also had closed scales that could be easily transformed into 0–1; exceptions were the BRIEF subscales and some CBCL subscales, which provided T‐scores (standardized scales with mean = 50 and SD = 10 for healthy individuals; Table 3).

**TABLE 3:** Descriptives from subsample 1's (*n* at most 80) external variables.

External variable	*N*	Scale	Mean	SD	Min	Max
VABS‐II, Grossmotor	80	0–1	.761	.151	.275	1.000
VABS‐II, Finemotor	80	0–1	.665	.224	.222	1.000
VABS‐II, Interpersonal Relationships	80	0–1	.541	.156	.276	.895
VABS‐II, Play and Leisure Time	80	0–1	.529	.199	.065	1.000
VABS‐II, Receptive Communication	80	0–1	.650	.166	.175	1.000
VABS‐II, Expressive Communication	80	0–1	.619	.236	.111	1.000
VABS‐II, 6/41 items of the "Personal" subscale	80	0–1	.853	.212	.000	1.000
VABS‐II, 35/41 items of the "Personal" subscale	80	0–1	.514	.216	.014	.943
VABS‐II, Domestic	80	0–1	.305	.194	.000	.771
VABS‐II, Community	80	0–1	.276	.203	.000	.795
VABS‐II, Written Communication	80	0–1	.306	.268	.000	.960
BRIEF, Emotional Regulation	65	T‐scores	56.05	13.14	37.00	89.00
CBCL, Internalization	76	T‐scores	54.36	9.53	31.00	77.00
CBCL, Attentional Problems	76	T‐scores	66.51	8.95	51.00	92.00
CARS, overall score	80	0–1	.225	.125	.033	.578
CBCL, 20/113 items, Aggressive Behavior	68	0–1	.275	.134	.050	.625
BRIEF, Inhibition	65	T‐scores	63.27	14.33	41.00	99.00
BRIEF, Shift	65	T‐scores	63.24	15.52	37.00	99.00
CBCL, Externalization	68	0–1	.206	.105	.030	.485

Abbreviations: BRIEF, Behavior Rating Inventory of Executive Function; CBCL, Child Behavior Checklist; Max, maximum; Min, minimum; SD, Standard Deviation; VABS, Vineland Adaptive Behavior Scales.

#### 2.6.2 Score transformations

GLM was applied to obtain *z*‐scores as standardized residuals, taking into account potentially relevant demographics as predictors. However, GLM cannot be run on closed‐scale dependent variables, so all 0–1 scores (Y_01_) were transformed^36^ into open scales by applying Logit[Y_01_] = *ln*[Y_01_ / (1 – Y_01_)]. To avoid infinities, Y_01_ = 0 and Y_01_ = 1 values were first replaced by .01 and .99. Unless otherwise stated, Logit[Y_01_] were used as dependent variables. The Age predictor (months) was asymmetrical (skewness = +1.08), hence potentially producing leverage effects in regression. We thus used *ln*(Age)_0_, the log‐transformed Age minus its average value (4.962). Sex and Nationality were recoded into numerical variables Sex_0_ and Nat_0_ (male = −.5, female = +.5; Italian = −.5, French = +.5).

#### 2.6.3 Standardization of the D‐DAND *z*‐scores

Capitalizing on the full dataset (*N* = 123), we standardized all D‐DAND measures. Subsample 2 (*n* = 43) was given D‐DAND twice, so we only took the first administration from it. Each GLM had Logit[Y_01_] as dependent variable, and *ln*(Age)_0_, Sex_0_, Nat_0_, and the Sex‐by‐Nationality interaction (Sex_0_ × Nat_0_) as predictors. The standardized residuals from these GLMs were the desired *z*‐scores (Equation 1).

(1) $$z=\;\left\{\text{Logit}\left[Y_{01}\right]–\left[i+\beta_{A}\;\ln{\left(\mathrm{Age}\right)}_{0}+\beta_{s}\;{\mathrm{Sex}}_{0}+\beta_{N}\;{\mathrm{Nat}}_{0}+\beta_{\mathrm{SN}}\;{\mathrm{Sex}}_{0}\;{\mathrm{Nat}}_{0}\right]\right\}/{\mathrm{SD}}_{\mathrm{res}}$$

In Equation (1), Logit[Y_01_] is the logit transformation of the patient's 0–1 D‐DAND score; *i* (intercept), *β*_A_, *β*_S_, *β*_N_, *β*_SN_, and SD_res_ (SD of the regression's residuals) are the parameters obtained from GLM; *ln*(Age)_0_, Sex_0_, and Nat_0_ are the patient's demographics.

“Per‐Week Awakenings” (D.19) was treated differently. It was markedly asymmetrical (Skewness = +3.16) with a peak at zero, so no transformation could normalize its shape and allow for GLM. Thus we ran a Generalized Linear Model (GzLM) with Tweedie distribution and log‐link, which is tailored exactly for that distributional shape. As a consequence, no *z*‐scores but only percentiles could be provided in this case.

#### 2.6.4 D‐DAND scores' reliability

Subsample 2's (*n* = 43) data provided test–retest reliability indices for all D‐DAND scores. Each reliability index was extracted by covarying out the effect of possible confounds (*ln*[Age]_0_, Sex_0_, Nat_0_, Sex_0_ × Nat_0_). Thus, a GLM was run using the D‐DAND score from the first administration as the main predictor, the four confounds (see above) as extra predictors, and the D‐DAND score from the second administration as the predicted variable. The square root of the partial eta‐squared of the effect of the first‐administration D‐DAND score is the reliability index (−1,1), free from distortion/inflation effects by the confounds.

#### 2.6.5 Significance of D‐DAND score changes

Once the reliability coefficient of a D‐DAND scale is available, a statistical test for the significance of a change across time can be run. This is based on the difference *D*_*z*_ between the *z*‐scores obtained from separate assessments of the same patient. The statistic is *D*_*z*_/(2–2*r*_xx_)^.5^, where *r*_xx_ is the reliability coefficient, and follows a standard normal distribution *Norm*(0, 1), under H_0_ of no change.

#### 2.6.6 Correlation with external variables

Subsample 1's (*n* = 80) data provided correlations between D‐DAND scores and the “external” variables (Table 3) that were coupled with them in terms of content. These helped illustrate the relationship of D‐DAND with widely used, but not DS‐tailored, scales. We applied the same procedure employed in the reliability analysis, but this time the predicted variable was a D‐DAND score, and the critical predictor was the external variable coupled with it, together with the four usual regressors, *ln*(Age)_0_, Sex_0_, Nat_0_, and Sex_0_ × Nat_0_. The square root of the partial eta‐squared of the effect of the external variable expresses the degree of association between the D‐DAND score and the external variable itself, free from distortions/inflations possibly produced by the other regressors.

To guarantee as close a semantic match as possible between the D‐DAND subscales and their coupled external variables, two items of the D.8 “Feeding” subdomain (“Swallowing” and “Appetite”) and two items of the D.12 “Academic Skills” subdomain (“Numbers and Counting” and “Calculation”) were excluded from the computation of the D scores.

## 3 RESULTS

Table 2 (left‐side columns) reports descriptives from the D‐DAND scores of the full sample (*N* = 123), which underwent the standardization analysis. All D‐DAND scores are reported on the 0–1 scale except for D.18 (Total Sleep Hours per Day), which is reported on the 0–24 scale. Table 3 reports descriptives from external variables (subsample 1, *n* = 80), which were missing for a few patients and scales (BRIEF and CBCL).

### 3.1 D‐DAND's standardization

Table 2 reports the results of GLMs on the full standardization sample (*N* = 123). Significant age‐related improvement was observed for fine motor abilities (D.3), personal/social autonomy (D.9, D.11), academic skills (D.12), and attention/motor control (D.15), whereas significant age‐related worsening emerged for energy/sport activity (D.2), internalizing behavior (D.14), and social–communication problems (D.16); daily sleep duration decreased with age (D.18). Sex, Nationality, and their interaction showed some sparse significant effects (see Table 2), which are of difficult interpretation (especially those concerning Nationality, which are likely to involve subtle translation‐related nuances); however, in the present work predictors are useful only insofar as they allow for better estimation of the residual variance, which in turn allows for accurate derivation of *z*‐scores. GzLM on weekly nocturnal awakenings (Tweedie distribution, log‐link) failed to detect any significant effects by the predictors (*ln*[Age]_0_, Nat_0_, Sex_0_, Sex_0_ × Nat_0_: Wald *χ* ^2^ = .296, 3.787, .014, 1.438; *p* = .587, .052, .907, .23).

### 3.2 D‐DAND's reliability

Test–retest reliability was obtained from subsample 2 (43 patients). The final reliability indices *r*_xx_ (Table 4) were Pearson partial correlation coefficients for subdomain scores D.1–D.18. Total Awakenings per Week (D.19) was an exception, with its markedly asymmetrical distribution (skewness = +3.16); given that GzLM found no significant effect by any of the predictors, we did not need to covary out the effects by confounds and could estimate a reliability index by simply computing the distribution‐free Spearman's Rho correlation between the two lists of raw awakening counts. This was very high, *Rho*_*xx*_ = .971.

**TABLE 4:** Reliability study (subsample 2, *n* = 43).

		Scale	D‐DAND first administration				D‐DAND second administration				Reliability	D_*z*_ significant if >	
			Mean	SD	Min	Max	Mean	SD	Min	Max	*r*_xx_	*α*=.1	*α*=.05
D.1	Grossmotor	0–1	.679	.188	.155	.969	.686	.184	.190	.979	.902	.728	.868
D.2	Energy and Sport Activity	0–1	.762	.244	.250	1.000	.692	.322	.000	1.000	.666	1.344	1.602
D.3	Finemotor	0–1	.726	.201	.147	1.000	.741	.203	.147	1.000	.928	.624	.744
D.4	Social Interaction	0–1	.608	.215	.097	1.000	.636	.213	.083	1.000	.952	.510	.607
D.5	Gaming	0–1	.547	.348	.000	1.000	.578	.353	.000	1.000	.767	1.123	1.338
D.6	Communication: comprehension	0–1	.645	.191	.250	1.000	.686	.205	.250	1.000	.574	1.518	1.809
D.7	Communication: production	0–1	.717	.224	.100	1.000	.713	.215	.100	1.000	.904	.721	.859
D.8	Feeding / Alimentation	0–1	.789	.133	.354	1.000	.825	.114	.563	1.000	.739	1.188	1.416
D.9	Personal Autonomies	0–1	.594	.299	.111	1.000	.603	.277	.111	1.000	.948	.530	.632
D.10	Domestic Autonomies	0–1	.398	.252	.000	1.000	.377	.221	.000	.889	.842	.925	1.102
D.11	Social autonomies	0–1	.314	.209	.000	.754	.341	.240	.000	1.000	.858	.877	1.044
D.12	Academic Skills	0–1	.416	.346	.000	1.000	.417	.339	.000	1.000	.970	.406	.484
D.13	Emotional Regulation	0–1	.576	.347	.000	1.000	.616	.329	.000	1.000	.868	.845	1.007
D.14	Internalizing Behavior	0–1	.814	.182	.250	1.000	.810	.179	.417	1.000	.860	.870	1.037
D.15	Attention and Motor Control	0–1	.586	.185	.125	.938	.616	.173	.125	.938	.590	1.489	1.775
D.16	Social/Communication Problems	0–1	.738	.214	.229	1.000	.756	.217	.146	1.000	.805	1.027	1.224
D.17	Externalizing Behavior	0–1	.384	.290	.000	1.000	.384	.252	.000	1.000	.815	1.001	1.192
D.18	Total Sleep Hours Per Day	0–24	10.30	1.33	6.50	13.00	10.22	1.42	7.00	13.50	.784	1.081	1.288
D.19	Total Awakenings Per Week	0‐	1.41	2.63	.00	10.00	1.35	2.51	.00	10.00	.971^a^		
D.Mot	Motor Abilities	0–1	.701	.170	.159	.982	.706	.170	.161	.982	.960	.465	.554
D.Lan	Language and Social Interaction	0–1	.641	.202	.119	.976	.659	.202	.113	.976	.968	.416	.496
D.Aut	Autonomies	0–1	.528	.172	.202	.835	.543	.162	.226	.867	.935	.593	.707
D.AcS	Academic Skills	0–1	.416	.346	.000	1.000	.417	.339	.000	1.000	.970	.406	.484
D.Emo	Emotional/Behavioral Regulation	0–1	.669	.121	.391	.923	.686	.129	.353	.942	.918	.666	.794
D.OS	Overall Score	0–1	.616	.145	.303	.857	.629	.143	.300	.874	.984	.294	.351

*Note*: Descriptives from D‐DAND subscales are reported, as well as reliability indices *r*_xx_ partialing out effects by confounds (Age, Sex, Nationality, Sex by Nationality). The last two columns report the smallest *z*‐score differences (*D*_*z*_) that are significant at the (two‐tailed) α levels .1 and .05. Single *z*‐scores are obtained by Equation 1 (see text). Abbreviations: D‐DAND, Dravet Disease‐Associated Neuropsychiatric Disorders; Max, maximum; Min, minimum; SD, Standard Deviation.

^a^ Spearman's Rho.

Overall, reliability coefficients *r*_xx_ were very high (median = .885; Table 4). The overall score D.OS had nearly perfect reliability (.984), showing excellent stability over 2 weeks. A similar pattern was found for most subscales. For instance, the Motor Abilities domain (D.Mot) had *r*_xx_ = .96, and the Emotional/Behavioral Regulation domain (D.Emo) had *r*_xx_ = .918. Exceptions were *r*_xx_ = .574 for D.6 and *r*_xx_ = .59 for D.15. Overall, these findings indicate that caregivers provided consistent reports and that the instrument yields stable measurements of patient status in the short term. D‐DAND's reliability is similar to that observed with classical scales.^23^, ^24^, ^25^, ^26^, ^27^, ^28^, ^29^, ^30^, ^31^, ^32^

### 3.3 Significance of D‐DAND changes across time

Reliability coefficients *r*_xx_ allow one to compare D‐DAND scores across time and decide whether a significant change took place. Change must be computed after having transformed single D‐DAND scores into *z*‐scores by means of Equation 1. A difference between *z*‐scores is significant at the α level if its absolute value is higher than the cutoff reported in the last two columns of Table 4.

### 3.4 Correlation of D‐DAND with external variables

Table 5 reports the correlations between each D‐DAND score and its coupled external variable, as derived from the GLM that partialed out the effects by other predictors. The expected direction of the correlation was deduced on grounds of the direction of pathology in the external scales (pathology was always toward lower values in D‐DAND).

**TABLE 5:** Correlations between D‐DAND scores and coupled external variables, controlling for the effects by confounds (Age, Sex, Nationality, Sex by Nationality).

External scale	DAND subdomain	*N*	Correlation		
			Expected direction	Estimated	Two‐tailed *p*
VABS‐II, Grossmotor	D.1 Grossmotor	80	+	.864	<.001
VABS‐II, Finemotor	D.3 Finemotor	80	+	.854	<.001
VABS‐II, Interpersonal Relationships	D.4 Social interaction	80	+	.781	<.001
VABS‐II, Play and Leisure Time	D.5 Gaming	80	+	.762	<.001
VABS‐II, Receptive Communication	D.6 Communication: comprehension	80	+	.592	<.001
VABS‐II, Expressive Communication	D.7 Communication: production	80	+	.728	<.001
VABS‐II, 6/41 items of the "Personal" subscale	D.8 Alimentation	80	+	.628	<.001
VABS‐II, 35/41 items of the "Personal" subscale	D.9 Personal autonomies	80	+	.836	<.001
VABS‐II, Domestic	D.10 Domestic autonomies	80	+	.804	<.001
VABS‐II, Community	D.11 Social autonomies	80	+	.858	<.001
VABS‐II, Written Communication	D.12 Academic Skills	80	+	.926	<.001
BRIEF, Emotional Regulation	D.13 Emotional regulation	65	−	−.228	.008
CBCL, Internalization	D.14 Internalizing behavior	76	−	−.110	.353
CBCL, Attentional Problems	D.15 Attention and motor control	76	−	−.290	.014
CARS, overall score	D.16 Social/communication problems	80	−	−.635	<.001
CBCL, 20/113 items, Aggressive Behavior	D.17 Externalizing behavior	68	−	−.226	.072
CARS, overall score	D.4 Social interaction	80	−	−.705	<.001
BRIEF, Inhibition	D.15 Attention and motor control	65	−	−.366	.004
BRIEF, Shift	D.16 Social/communication problems	65	−	−.311	.015
CBCL, Externalization	D.17 Externalizing behavior	68	−	−.207	.101

*Note*: The estimated Pearson correlations are reported together with their two‐tailed *p*‐values. Abbreviations: BRIEF, Behavior Rating Inventory of Executive Function; CARS, Childhood Autism Rating Scale; CBCL, Child Behavior Checklist; D‐DAND, Dravet Disease‐Associated Neuropsychiatric Disorders; VABS, Vineland Adaptive Behavior Scales.

Correlations between D‐DAND scores and external measures (VABS subscales and CARS) were all in the expected direction but varied widely in magnitude (|*r*| ranged from .11 to .926; Table 5), indicating both shared and distinct measurement content. Domains closely aligned with standard constructs—particularly motor and adaptive/daily living skills (D.1–D.11)—showed strong convergence with VABS‐II, with especially high agreement when averaged across matched subdomains (mean D‐DAND D.1–D.11 vs. mean corresponding VABS‐II subscales: Pearson *r* = .956) and several domain‐level couplings in the *r* ≈ .7–.9 range (e.g., Social Interaction D.4 with VABS‐II Socialization; Communication–Production D.7 with VABS‐II Expressive Communication). In contrast, weaker or absent correlations for areas only partially captured by external instruments—such as feeding/alimentation (D.8) and more nuanced autism‐related behaviors (D.16) relative to CARS—support the notion that D‐DAND adds DS‐relevant information beyond conventional scales.

## 4 DISCUSSION

### 4.1 Aims and main findings

Dravet Syndrome (DS) presents a complex clinical picture in which seizures represent only one component.^2^ Comorbid developmental delay, behavioral difficulties, and problems regarding sleep and feeding, can be as impactful as seizures on patients' and families' lives.^16^ Moreover, seizures—and especially status epilepticus—often lessen in adolescence and early adulthood, whereas intellectual disability and behavioral and other comorbidities become increasingly prominent. We aimed at providing a new tool that, filling a gap in the literature, allows for quantification of the main behavioral and functional features of DS patients. This tool allows overcoming the limits of previously used tools, such as lack of specificity for DS, ceiling/floor effects, and time for administration. In doing so, we wished to provide measures with a clear clinical meaning, *z*‐scores. Through several sessions, we elicited empirical and clinical knowledge from experts and literature, and converged toward a tool, D‐DAND, which was administered to 123 DS caregivers and provided very stable responses in a subsample of them over a short time period (test–retest *r*_xx_ = .984 for the overall *z*‐score). This high stability supports the use of D‐DAND for longitudinal tracking, as any substantial change in *z‐*scores would express an evolution that is beyond what can be explained by age‐related effects (which are partialed out by *z*‐scores) or measurement error (see Table 4 for cutoffs of significant *z*‐score differences). Despite this possibility, however, we do not have direct empirical evidence of individual long‐term trajectories yet. We compared D‐DAND, which has been tailored to use with DS patients, with other, “external” scales that are commonly used in everyday clinical work but that were developed for different purposes/populations (e.g., individuals without intellectual disability). We expected to find good agreement in some scales and bad agreement in others, exactly because the external scales had been developed to quantify behavioral features that do not always overlap the critical ones for DS. Consistently with this prediction, correlations varied very widely, from ~.1 up to ~.9. For instance, patients scoring higher in D‐DAND adaptive domains also had higher VABS‐II scores, but some CBCL scores barely correlated with the “corresponding” D‐DAND subscales.

These near‐zero correlations are indirect proof that a new scale was needed, and that those domains likely represent observational areas (e.g., feeding and behavioral issues) for which the existing tools are inadequate. For instance, D‐DAND quantifies persistent language and social–communication difficulties irrespective of seizure control and includes motor items sensitive to gait/coordination issues that may emerge later and further decline with age; it also addresses issues with feeding, sleep, and behavioral anomalies that are often overlooked. D‐DAND was not built just to provide a shorter version of existing tools, but rather to extend the holistic assessment into areas unique to DS as a developmental and epileptic encephalopathy prototype, and to easily assess complex behavioral dimensions. D‐DAND groups different aspects of the disease's morbidity into a single assessment, which is agile, time‐friendly, and easy to address and to interpret. Generally, both caregivers and administrators found the interview easy to understand and relevant; as a result, the degree of collaboration was high. No difficulties were reported during test administration. Families appreciated the opportunity to discuss a broad array of concerns, noting that this is at variance with the typical clinical colloquia, which often focus just on seizures. On the other side, clinicians who used D‐DAND found its summary profile useful to quickly gauge a patient's status across multiple domains.

### 4.2 Implications for practice

By estimating the patient's global status and multiaxial comorbidities, the D‐DAND interview helps neurologists and interdisciplinary teams to manage DS and to orient rehabilitation and further assessments. It may function as a fast screening/triage tool to identify patients who warrant in‐depth (neuro)psychological evaluation and targeted investigation of sleep and feeding disturbances; for example, abnormal sleep‐related scores could prompt referral to sleep specialists or behavioral sleep interventions that might otherwise be deprioritized in seizure‐focused care. D‐DAND is also suited for longitudinal follow‐up, enabling clinicians to document developmental progress, plateauing, or regression, and to frame differential hypotheses regarding drivers of change (e.g., seizure burden, treatment effects, versus disease natural history), thereby informing proactive intervention planning. Its caregiver‐interview format—codeveloped by experts, advocacy organizations, caregivers, and patients—may additionally enhance communication by aligning the assessment with family‐reported priorities. Finally, the scale's brevity (63 items) allows for administration in one standard appointment, potentially in telemedicine modality,^37^ thus facilitating systematic integration of functional monitoring with electroclinical evaluations and supporting a holistic, anticipatory care model in DS in which nonseizure outcomes are considered alongside seizure‐related measures.

### 4.3 Limitations

Our sample size (*N* = 123), although definitely respectable for a rare disease like DS, is still suboptimal for many fine‐grain analyses, such as factor analysis. The hierarchy that groups the 60 items into 19 subdomains and five domains (Figure 1) is just an initial proposal, grounded in the early phases of expert panel discussion, in which the practical need emerged to characterize the D‐DAND's output in terms of areas of intervention rather than areas of neurocognitive impairment. The hierarchy was thus conceived of as a pragmatic way to obtain a manageable summary of the patient's profile for clinicians and caregivers; items belonging to the same domain were thought by experts to be clinically relevant to a homogeneous area of care or intervention—for example, issues with school/education (the “Academic Skills” domain)—rather obviously reflecting diverse neuropsychological processes and deficits (reading, writing, number processing, etc.). Future work with larger samples might well suggest changes in such hierarchy, on theoretical, practical, or statistical grounds.

Our sample was cross‐sectional; thus, although we showed high test–retest reliability, which guarantees high sensitivity to change, we did not directly assess the responsiveness of D‐DAND scores over longer time lags or after intervention. Future longitudinal studies should evaluate how D‐DAND scores evolve over months/years, and whether changes correlate with clinical events (e.g., occurrence of status epilepticus or new treatments or interventions). Additionally, cultural or language differences could influence specific items; we combined Italian and French data after careful translation/back‐translation of the scale, but subtle differences in caregiver interpretation are likely to exist and may explain some of the significant effects involving Nationality (Table 1). Ongoing validation in other countries and languages would strengthen generalizability. Given that D‐DAND was proposed exactly because of the lack of a tool tailored to DS, comparison with other tools tailored to the same population was impossible at this stage. We filled this gap by grounding every item in the experts' consensus and in the analysis of relevant literature. Future use will reveal areas for refinement; we envision D‐DAND to be adjusted as more data accumulates (e.g., possibly shortening the scale or adjusting item selection).

### 4.4 What D‐DAND does and what it does not do

To interpret D‐DAND outputs correctly, it is important to highlight that this scale is a quantification and monitoring tool. D‐DAND signals the presence and degree of specific problems with a DS patient and allows one to detect statistically significant changes in those issues across time. D‐DAND does not compare the competences of a DS patient to those of typically developing individuals. Given the frequently severe deficits of DS patients, such comparison would not be fully informative, as the latter population is virtually always at a ceiling on the abilities that need evaluation in DS. D‐DAND investigates the range of ability that is typically not covered in the normal population—the range between moderate and severe deficit; it is in this region of performance that quantification is clinically and practically relevant. It is also important to highlight that D‐DAND is a tool for quantifying /measuring observable behaviors, and not latent constructs. Domains and subdomains are collections of items that constitute areas of practical intervention, and not behavioral variables entailing a single latent construct. For instance, the items concerning: use of spoon, fork, knife; chewing, swallowing, level of appetite (D.8); personal hygiene, dressing, sphincter control (D.9); awareness of dangers, household chores, food preparation (D.10); use of phone/tablet/computer, temporal orientation, money use (D.11), all belong to the area of intervention related to “Autonomies” (the name of the domain), but certainly do not reflect a single, neuropsychological latent process.

### 4.5 Future directions

We plan to increase sample size to be able to perform more extensive psychometric testing, including factor analysis and item response analysis. This would allow us to learn whether some items can be dropped or weighted differently. We will also explore whether D‐DAND scores correlate with seizure burden or genotype (preliminary data from other studies suggest that developmental outcome in DS is only partially dependent on seizure control). This could provide insight into how much improving seizures alone addresses (or fails to address) the broader challenges in DS. Further steps will concern translation into other languages and spreading of the tool through reference centers and networks like EpiCARE and patients' advocacy groups.

## 5 CONCLUSIONS

This initial study suggests that D‐DAND is a psychometrically sound and clinically useful tool for capturing the full range of developmental and neuropsychiatric problems in DS. This direction aligns with recent evidence that current nonseizure outcome tools are heterogeneous and frequently inadequate—especially for cognitive monitoring—and with calls for Dravet‐validated, Dravet‐specific cognitive measures.^38^ D‐DAND enables clinicians and caregivers to “see the whole picture” of the patient, facilitating comprehensive care. Furthermore, because it is standardized, low‐burden, and Dravet‐specific, D‐DAND is well suited for follow‐ups; it supports consistent baseline characterization and stratification, offers a caregiver‐reported outcome for nonseizure effects, and enables longitudinal monitoring alongside seizure agenda and metrics. D‐DAND might improve care and strengthen natural history readouts by looking beyond seizures. Use of this scale should help validate the detection of minimal meaningful changes in morbidities that are critical for the follow‐up of new therapies and interventions in this population.

## AUTHOR CONTRIBUTIONS

Bernardo Dalla Bernardina, Francesca Offredi, Tommaso Lo Barco, and Francesca Darra contributed to the development and clinical testing of the DAND scale at the Child Neuropsychiatry Unit, Azienda Ospedaliera Universitaria Integrata of Verona, Italy. Lisa Ouss, Delphine Breuillard, Mathilde Cozzo, and Rima Nabbout contributed to the development and clinical testing of the DAND scale at the Child Neuropsychiatry Unit, Necker‐Enfants Malades Hospital, Assistance Publique–Hôpitaux de Paris, Paris, France. Alessio Toraldo performed the statistical analyses. Isabella Brambilla coordinated the project, provided support throughout the study, and contributed as a family representative. Bernardo Dalla Bernardina, Francesca Offredi, Alessio Toraldo, Isabella Brambilla, and Rima Nabbout drafted the manuscript and revised it critically for important intellectual content, with input from all authors. All authors approved the final version of the manuscript and agree to be accountable for all aspects of the work.

## CONFLICT OF INTEREST STATEMENT

None of the authors has any conflict of interest related to the present work to disclose.

## ETHICS STATEMENT

We confirm that we have read the Journal's position on issues involved in ethical publication and affirm that this report is consistent with those guidelines.

## Supporting information


**FIGURE S1.** Key phases in the development of D‐DAND: domain identification, expert panel refinement, structural revisions, pilot testing in Italy and France, caregiver feedback, and final tool integration.

## Data Availability

The authors confirm that the data supporting the findings of this study are available within the main text and the supporting information. The data that support the findings of this study are available from the corresponding author upon reasonable request.
