# Supplementary material for: Introducing the D‐DAND scale: Development of a comprehensive caregiver‐administered tool for Dravet syndrome comorbidities
Source: Epilepsia. 2026 Aug 18;67(10):5325–39. doi: 10.1002/epi.70399 (PMC13675760; doi:10.1002/epi.70399)
Supplement: Supplementary file 1 [file EPI-67-5325-s001.docx]

**Supplementary information**

**Introducing the D-DAND scale: development of a comprehensive caregiver-administered tool for Dravet syndrome comorbidities**

Bernardo Dalla Bernardina^1^, Francesca Offredi^1,2^, Alessio Toraldo^3,4^, Lisa Ouss^5^, Delphine Breuillard^5^, Mathilde Cozzo^5^, Tommaso Lo Barco^1^, Isabella Brambilla^1,6^, Francesca Darra^1,2,7^, Rima Nabbout^5,8^

^1^ Research Center for Pediatric Epilepsies (CREP), Azienda Ospedaliera Universitaria Integrata (AOUI) of Verona Italy

^2^ Child Neuropsychiatry Unit, Azienda Ospedaliera Universitaria Integrata (AOUI) of Verona Italy, Full Member of European Reference Network EpiCARE

^3^ Department of Brain and Behavioural Sciences, University of Pavia, Pavia, Italy

^4^ Milan Center for Neuroscience (NeuroMI)

^5^ Reference Center for Rare Epilepsies, Department of Pediatric Neurology, Necker Enfants Malades Hospital, APHP, Full Member of European Reference Network EpiCARE, Université Paris Cité, Paris, France

^6^ Dravet Italia Onlus (Fondazione Dravet ETS) , Member of the ERN EpiCARE Patient Advocates Group, Verona, Italy.

^7^ Child Neuropsychiatry Section, Department of Engineering for Innovation Medicine University of Verona, Italy

^8^ Institut Imagine, INSERM U1163, Paris, France

**Corresponding author**

**Alessio Toraldo
e-mail:** alessio.toraldo@unipv.it

**ORCID**

**Bernardo Dalla Bernardina**	0000-0002-8740-4686

**Francesca Offredi**	0009-0003-8420-611X

**Alessio Toraldo**	0000-0001-6480-4751

**Tommaso Lo Barco**	0000-0002-3103-230X

**Isabella Brambilla**	0000-0002-9860-8246

**Delphine Breuillard**	0009-0000-6317-900X

**Francesca Darra**	0000-0002-1062-8438

**Rima Nabbout**	0000-0001-5877-4074

**Lisa Ouss**	0000-0002-5266-9836

**Mathilde Cozzo**	0009-0003-9744-2349

**SUPPLEMENTARY METHODS**

***Supplementary Figure 1.*** *Key phases in the development of D-DAND: domain identification, expert panel refinement, structural revisions, pilot testing in Italy and France, caregiver feedback, and final tool integration.*
